# *Avena sativa* as a Multifunctional Tool for Phytoremediation and Bioenergy Production in Sulfentrazone Contaminated Soils

**DOI:** 10.3390/jox15030087

**Published:** 2025-06-04

**Authors:** Caique Menezes de Abreu, Guilherme Henrique Fernandes Carneiro, Márcia Regina da Costa, Gabriela Madureira Barroso, Tayna Sousa Duque, Joice Mariana Santos Silva, José Barbosa dos Santos

**Affiliations:** Department of Agronomy, Federal University of Jequitinhonha and Mucuri Valleys, Diamantina 39100-000, MG, Brazil; menezes.abreu@ufvjm.edu.br (C.M.d.A.); henrique.guilherme@ufvjm.edu.br (G.H.F.C.); marcia.costa@ufvjm.edu.br (M.R.d.C.); gabriela.madureira@ufvjm.edu.br (G.M.B.); joice.mariana@ufvjm.edu.br (J.M.S.S.); jbarbosa@ufvjm.edu.br (J.B.d.S.)

**Keywords:** agriculture, herbicide, microbial biodiversity, remediation, soil contamination, sustainability

## Abstract

Phytoremediation using *Avena sativa* offers a sustainable strategy for mitigating sulfentrazone contamination while integrating bioenergy production. This study proposes an analysis of the bioenergy potential and the microbial metagenomic profile associated with *Avena sativa* in the presence and absence of sulfentrazone, aiming at the synergistic bioprospecting of microbial communities capable of biodegradation and remediation of contaminated environments. Using a randomized block design, we evaluated the bioenergy potential and rhizospheric microbial dynamics of *A. sativa* in soils with and without sulfentrazone (600 g ha^−1^). Herbicide residues were quantified via UHPLC-MS/MS, and metagenomic profiles were obtained through 16S rRNA gene and ITS region sequencing to assess shifts in rhizospheric microbiota. Microbial diversity was analyzed using the Shannon and Gini–Simpson Indices, complemented by Principal Component Analysis (PCA). Bioenergy yields (biogas and ethanol) were estimated based on plant biomass. Over 80 days, the cultivation of *A. sativa* promoted a 19.7% dissipation of sulfentrazone, associated with rhizospheric enrichment of plant growth-promoting taxa (*Bradyrhizobium*, *Rhodococcus*, and *Trichoderma*), which increased by 68% compared to uncontaminated soils. Contaminated soils exhibited reduced microbial diversity (Gini–Simpson Index = 0.7), with a predominance of *Actinobacteria* and *Ascomycota*, suggesting adaptive specialization. Despite herbicide-induced stress (39.3% reduction in plant height and 60% reduction in grain yield), the biomass demonstrated considerable bioenergy potential: 340.6 m^3^ ha^−1^ of biogas and 284.4 L ha^−1^ of ethanol. The findings highlight the dual role of *A. sativa* in soil rehabilitation and renewable energy systems, supported by plant–microbe synergies. Scalability challenges and regulatory gaps in ecotoxicological assessments were identified, reinforcing the need to optimize microbial consortia and implement region-specific management strategies. These results support the integration of phytoremediation into circular bioeconomy models, balancing ecological recovery with agricultural productivity. Future research should focus on microbial genetic pathways, field-scale validation, and the development of regulatory frameworks to advance this green technology in global soil remediation efforts.

## 1. Introduction

Globally, herbicides represent the most widely used class of pesticides in agriculture, raising increasing concerns among the scientific community, civil society, and governmental agencies [1]. Among these compounds, sulfentrazone is a pre-emergent, conditionally selective herbicide with systemic action. It belongs to the triazolinone chemical group and acts primarily by inhibiting the plant’s enzyme protoporphyrinogen oxidase (PROTOX).

This herbicide is effective in controlling grasses and broadleaf weeds across various crops and is also used in the management of resistant species such as *Amaranthus palmeri* in soybean cultivation [2,3]. It has high water solubility and a half-life exceeding 120 days in aerobic and anaerobic soils [4]. Due to its physicochemical mobility, sulfentrazone exerts intense selective pressure on populations of resistant weeds, particularly in high-production environments.

The chemical interactions of sulfentrazone with the soil are directly linked to the degradation of its physicochemical properties, including the reduction in cohesion and the internal friction angle, thereby compromising the structural stability of the soil [5]. In addition to direct effects on the microbiota, the persistence of these compounds imposes pressure on agricultural ecosystems, demanding integrated remediation strategies [6,7]. Among these technologies, phytoremediation stands out, employing tolerant plants such as *Avena sativa* [8] for the removal of soil salts [9], heavy metals like Pb and Th [10], Cr, Zn, Cu, and Ni [11], and herbicides [12].

In addition to its phytoremediating potential, *A. sativa* is an energy crop of interest, with applications in ethanol and biogas production [13]. Its hemicelluloses constitute approximately one-third of the cell wall and can be used as raw material for biofuels and other industrial applications. The genes responsible for hemicellulose synthesis have already been extensively characterized in their structural, functional, and evolutionary aspects [14].

An innovative approach proposed in this research is the study of the impact of sulfentrazone on the microbial dynamics of the soil, associated with the conditioning of *A. sativa* for enhanced performance in soil decontamination and bioenergy generation. Phytoremediation, as a green technology, represents a promising utility model by integrating the degradative potential of microorganisms with plant species capable of tolerating herbicide residues [15].

Plant–microorganism interactions intensify in the rhizosphere zone, benefiting both plants and microbial communities by facilitating the degradation of decomposition by-products of sulfentrazone. This process can be promoted by plants such as *Canavalia ensiformis* [16], microorganisms, or through their combined action. Herbicides with high toxicity and residual effects require alternative techniques for the adsorption and removal of undesirable compounds or recovering soil-beneficial components. Although herbicides may provide carbon sources for microbial growth and consequently confer a competitive advantage to certain microorganisms [17], little is known about the dynamics of native microbiota and their potential for herbicide biodegradation.

Among the sulfentrazone-degrading microorganisms already identified, *Nocardia brasiliensis* and *Penicillium* sp. are noteworthy [18]. When associated with phytoremediating plants, these microorganisms may contribute to developing promising soil decontamination technologies in areas subjected to prolonged herbicide application. Several plant species have already been reported as potential sulfentrazone remediators, mostly legumes capable of associating with specific microbial groups [19].

During the phytoremediation process, it is expected that microbial communities with high taxonomic and functional diversity will promote the growth of remediating plants and accelerate the degradation of xenobiotics, thereby mitigating the deleterious effects of these compounds on human and environmental health [20]. Plants have demonstrated the ability to optimize bacterial communities in soils contaminated with sulfentrazone [16]. To enhance this technology, functional genomics and the integration of genetic and productive data are essential for constructing microbiomes highly adapted to agricultural environments. This approach harnesses cellular and molecular processes, transforming them into high-value-added products and innovations.

In this context, the objectives of this study are to (i) analyze the microbial metagenomic profile associated with *A. sativa* in soils contaminated and uncontaminated with sulfentrazone, identifying key communities involved in synergistic biodegradation; (ii) evaluate the bioenergy potential of *A. sativa* under herbicide stress, correlating biomass production with remediation efficiency; and (iii) explore the relationship between microstructural changes in the soil induced by contaminants and the effectiveness of green remediation technologies, based on integrated microbiological and mechanical data.

Thus, the present study proposes the analysis of the bioenergy potential and the microbial metagenomic profile associated with *Avena sativa* in the presence and absence of sulfentrazone, aiming at the synergistic bioprospecting of communities capable of biodegradation and remediation of contaminated environments.

## 2. Materials and Methods

The experiment was carried out in an experimental area characterized as Neosol Quartzarenico Órtico with sandy loam texture (Brazilian soil classification system) equivalent to Entisols soils (USDA soil taxonomy) [21,22], with a pH of 5.2, containing 1.2 cmolc dm^−3^ of Ca^2+^, 36 mg dm^−3^ of K, and 1.6 dag kg^−1^ of organic matter, located at latitude 18°10′ S and longitude 43°30′ W, with an altitude of 1388 m, and a subtropical highland climate—*Cwb*. The experimental design was randomized blocks with three replicates and four treatments: S0A1—*Avena sativa* cultivation in an area with 0.0 g ha^−1^ of sulfentrazone (2′,4’-dichloro-5’-(4-difluoromethyl-4,5-dihydro-3-methyl-5-oxo-1H-1,2,4-triazol-1-yl) ethanesulfonanilide); S1A0—absence of *A. sativa* cultivation in an area with a commercial dose of 600 g ha^−1^ of sulfentrazone; S1A1—*A. sativa* cultivation in an area with a dose of 600 g ha^−1^ of sulfentrazone; No0—undisturbed soil of the Cerrado biome. The species *Avena sativa* (cultivar: URS-Taura/BRSEE-Ds^®^) was chosen because of its potential for remediation in areas with herbicides.

After soil preparation and fertilization, the recommended practices for cover crop application, as outlined by Ghosh et al. (2014) [23], were followed to improve soil fertility and increase organic matter content. Based on standard agronomic practices, the herbicide dose was recommended for the main agricultural and forestry crops. For application, a backpack sprayer equipped with a crossbar containing four nozzles with a 110° angle and a flow rate of 0.15 gallons per minute was used, delivering 150.0 L ha^1^ of solution. The predetermined plots were sown with the species *A. sativa* three days after application, allowing for adequate dissipation of the herbicide’s immediate effects on the soil. The planting density was 7.0 g seeds/m^2^, with experimental plots measuring 32.5 m^2^ (5.0 × 6.5 m) [24].

### 2.1. Agronomic Performance and Phenological Characterization of Avena sativa

To evaluate the agronomic performance and phenotypic traits of *Avena sativa* cultivated under different soil contamination conditions, plant samples were collected from an area of 0.25 m^2^ per experimental plot 80 days after herbicide application. Plant height was measured using a graduated scale, and aboveground dry biomass was determined after drying the material in a forced-air oven at a temperature of 65 °C until constant weight.

The thousand-grain weight (TGW) was estimated from eight subsamples of 100 grains each, collected from 1 m^2^ of harvested area per plot. The determination followed the procedures outlined in the Seed Analysis Rules [25]. Grain yield was calculated based on processed grain mass, expressed in kg m^2^ and extrapolated to kg ha^−1^.

Ethanol production potential (L ha^−1^) was estimated based on the grain yield, while biogas production potential (m^3^ ha^−1^) was inferred from the dry mass of the aboveground biomass accumulated during the phytoremediation cycle.

The data were subjected to normality, homogeneity, and additivity tests. After meeting the assumptions, the data were analyzed by analysis of variance (ANOVA), and the means were compared by Tukey’s test at a 5% significance level.

### 2.2. Microbial Community Metagenomics

Three *A. sativa* plants per plot were sampled eighty days after herbicide application. The root systems were carefully removed, and rhizosphere soil was collected and stored at −80 °C for subsequent metagenomic analysis. Total metagenomic DNA was extracted and purified, and sequencing was performed on the Illumina HiSeq 2000 platform (Illumina, San Diego, CA, USA), targeting the 16S rRNA and ITS gene regions.

Raw sequence reads were pre-filtered using the Readfq v8 tool (https://github.com/cjfields/readfq, accessed on 22 January 2024) to eliminate low-quality reads and those with less than 10.0% high-quality bases.

Subsequently, the high-quality sequences of each sample were assembled (MEGAHIT), generating “Scaffolds” with fragments greater than or equal to 500 bp. High-quality reads were assembled using MEGAHIT (L3 Bioinformatics Limited, Hong Kong, China) to generate scaffolds ≥500 bp in length. Taxonomic annotation was performed by comparing the sequences to genomic databases of bacteria, fungi, and archaea using the BLASTx algorithm.

Microbial identification and relative quantification were based on species-level alignments to curated fungal and bacterial databases. Relative abundance (%) was calculated as the proportion of each taxon within its respective domain. Dominance was estimated using Gini–Simpson Indices [26], and richness was quantified by the number of species present in the sample, according to Chao1. Species diversity was quantified based on the number of species in the samples using the Shannon Index [27]. In addition, to indicate the distribution of the different species in the sample, we used the Pielou Index to determine evenness [28]. Comparative analyses of diversity indices among treatments were performed using a non-parametric Z test, with the No0 treatment (undisturbed soil from the Cerrado biome) serving as the ecological equilibrium baseline.

Multivariate analysis was conducted in R v4.4.1. Principal Component Analysis (PCA) was applied to microbial class abundance data to assess shifts in community structure. Significant groupings were identified based on correlation thresholds above 0.9 or below −0.9. Pearson correlation networks (thresholds > 0.6 or <−0.6) were used to examine the association between microbial orders and phytoremediation conditions, visualized through neural network graphs.

To evaluate the functional potential of the rhizosphere microbiome, bacteria and fungi with known plant growth-promoting traits were identified based on evidence from the literature. These functions included biological nitrogen fixation, nutrient solubilization, biocontrol activity (insecticidal, nematicidal, bactericidal, and fungicidal), phytohormone production, and siderophore synthesis. The relative abundance of functional taxa was calculated and compared across treatments using the Z test, referencing the No0 condition as a natural equilibrium standard.

### 2.3. Herbicide Residue Analysis by UHPLC MS/MS

Eighty days after herbicide application, soil samples from treatments S1A0 and S1A1 were collected to analyze sulfentrazone residues. Extraction was carried out using a modified QuEChERS method, as described by Brondi et al. (2011) [29]. The quantification and identification of residues were performed using ultra-high-performance liquid chromatography coupled with tandem mass spectrometry (UHPLC-MS/MS).

Prior to analysis, the samples were diluted 1:5 in ultrapure water. Analytical determinations were conducted using a Waters UHPLC-MS/MS system (Waters, Milford, MA, USA) equipped with a Xevo TQ triple quadrupole mass detector (Waters, Milford, MA, USA), an electrospray ionization (ESI) source/interface, a nitrogen generator, and a binary solvent delivery system for high-pressure gradient elution. Chromatographic separation was achieved using an Acquity UPLC^®^ BEH C18 analytical column (50 × 2.1 mm, 1.7 μm; Waters, Milford, MA, USA). Data were acquired and processed using MassLynx v4.1 software (Waters, Milford, MA, USA).

The compounds were quantified using the selected reaction monitoring (SRM) mode. The mobile phase consisted of (A) water and (B) methanol, both containing 5 mmol L^−1^ of ammonium formate and 0.1% formic acid (*v*/*v*), in a 98:2 (*v*/*v*) proportion for the aqueous phase. The flow rate was set at 0.225 mL min^−1^, and the injection volume was 10 µL, according to the protocol described by Kemmerich (2017) [30].

For qualitative analysis, herbicide dissipation was estimated based on each treatment’s average signal intensity (peak area). The results in the figures are expressed as means ± standard error of the mean (SEM).

## 3. Results

The remediation of soil contaminated with sulfentrazone was evaluated using *Avena sativa* plants at a stand density of 198.3 plants/m^2^ over 80 days. It was observed that the residual concentration of sulfentrazone was reduced from 0.066 ± 0.007 mg kg^−1^ in S1A0 to 0.053 ± 0.007 mg kg^−1^ in S1A1, resulting in a dissipation of 19.7% of the molecule in the presence of *A. sativa* (Supplementary Material S1).

*Avena sativa* showed satisfactory vegetative and reproductive growth both in the presence and absence of the herbicide dose. However, the height of plants undergoing phytoremediation in areas S1A1 was reduced by 39.3% compared to plants grown in areas without the presence of the molecule (S0A1) (Table 1).

The plants’ dry mass (MSPA) aerial part significantly differed between the S0A1 and S1A1 treatments. The accumulation of MSPA of the plants subjected to the phytoremediation system with sulfentrazone was 68.43% lower than the biomass production in the absence of the molecule, indicating that the plant is tolerant and that its vegetative material can be used for bioenergy production (Table 1). The biomass production of *A. sativa* in the soil decontamination system would result in an estimated biogas production of 340.6 m^3^ ha^1^.

The average grain weight (AWW) of *A. sativa* plants was influenced by the presence of the herbicide molecule in the soil. Plants subjected to the phytoremediation process reached the phenological reproduction stage, producing a grain mass 39.0% lower than the AWW of plants in areas without contamination by the herbicide molecule (S0A1) (Table 1).

The sulfentrazone residue reduced grain yield by 60.0% compared to areas without the herbicide molecule. The production of *A. sativa* was, on average, 2368.36 kg ha^−1^ in areas without contamination, while in regions undergoing phytoremediation with sulfentrazone, it was 947.86 kg ha^−1^ (Table 1). Bioethanol from *A. sativa* grains grown in the soil decontamination would produce 284.4 L ha^−1^ of ethanol.

**Figure 1:**
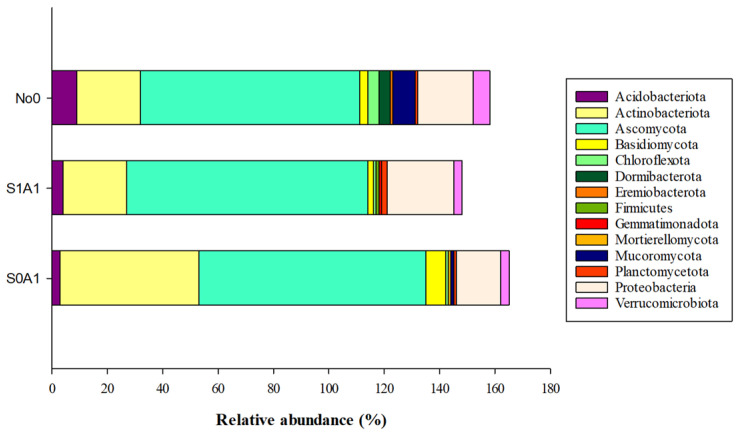
Relative abundance (%) of microbial phyla in typical areas of the Cerrado and sulfentrazone decontamination management using *Avena sativa*. S0A1—*Avena sativa* cultivation in an area with 0.0 g ha^−1^ of sulfentrazone; S1A1—*A. sativa* cultivation in an area with 600 g ha^−1^ of sulfentrazone; No0—Typical soil of the Cerrado biome.

The growth and development of the plants used in the phytoremediation process were influenced by the residue of the molecule, correlated with the modulation of the soil microbiota, in which the distribution of the relative abundance of the microbial phyla varied according to the environment. The most frequent phyla were *Ascomycota*, *Actinobacteriota*, *Proteobacteriota*, *Acidobacteriota*, and *Verrucomicrobiota* in areas No0, S0A1, and S1A1 (Figure 1). On the other hand, the phyla *Mucoromycota*, *Dormibacteriota*, and *Chloroflexota* were observed in less anthropized environments (No0).

**Figure 2:**
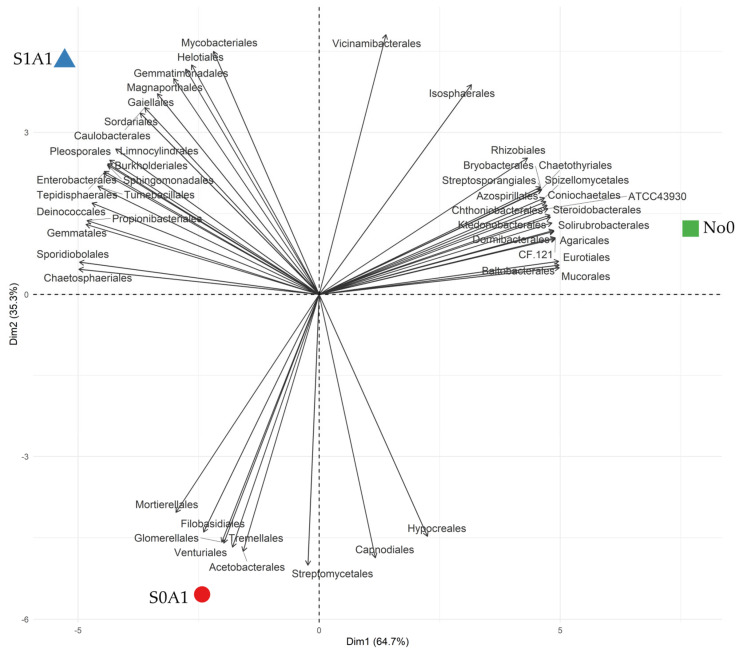
Principal Component Analysis (PCA) in residual sulfentrazone areas. S0A1—*Avena sativa* cultivation in an area with 0.0 g ha^−1^ of sulfentrazone; S1A1—*A. sativa* cultivation in an area with 600 g ha^−1^ of sulfentrazone; No0—Typical soil of the Cerrado biome.

Interspecific interactions are perceived in environmental changes. The PCA of soil phytoremediation with *A. sativa* highlighted the relationships between the abundance values of microbial phyla and the management of herbicide decontamination in the Cerrado, forming three phylogenetic groups (Figure 2). The populations that coexisted in the community in each treatment were selected by introducing the phytoremediator and/or herbicide, as they were better adapted to this new condition imposed on the environment. The orders of microorganisms responded satisfactorily to the biotic and abiotic pressure imposed by agricultural soil disturbance, even with the application of sulfentrazone and the cultivation of *A. sativa* (Figure 2).

The typical soil samples from Cerrado (No0) formed cluster I, where there was a greater correlation between 21 microbial orders, representing 43.7% of the relative abundance. The predominant orders were *Agaricales*, Indeterminate_A, *Azospirillales*, *Baltobacterales*, *Bryobacterales*, *Burkholderiales*, Indeterminate_B, *Chthoniobacterales*, *Coniochaetales*, *Dormibacterales*, *Isosphaerales*, *Ktedonobacterales*, *Mucorales*, *Rhizobiales*, *Solirubrobacterales*, *Sphingomonadales*, *Spizellomycetales*, *Steroidobacterales*, *Streptosporangiales,* and *Vicinamibacterales* (Figure 2).

Cluster II, representing 41.7% of the orders, was mainly composed of *Burkholderiales*, *Caulobacterales*, *Chaetosphaeriales*, *Deinococcales*, *Enterobacterales*, *Eurotiales*, *Filobasidiales*, *Gaiellales*, *Gemmatales*, *Gemmatimonadales*, *Helotiales*, *Limnocylindrales*, *Magnaporthales*, *Mycobacteriales*, *Pleosporales*, *Propionibacteriales*, *Sordariales*, *Sporidiobolales*, *Tepidisphaerales*, and *Tumebacillales*, presenting a higher correlation and frequency in anthropized environments (S1A1) when compared to typical soil (No0) (Figure 2).

The third cluster, with 14.6% of the orders, was formed by *Acetobacterales*, *Capnodiales*, *Filobasidiales*, *Glomerellales*, *Hypocreales*, *Mortierellales*, *Sordariales*, *Streptomycetales*, *Tremellales*, and *Venturiales*. These orders showed a high correlation with the activity of *Avena sativa*, and, in the presence of the herbicide molecule, a tendency for a reduction in the abundance of these organisms was observed (Figure 2).

The distribution of species within each environment was equitable. Biological activity in areas No0, S0A1, and S1A1 presented similar uniformity profiles, with deviations of up to ±0.3 (Table 2).

**Figure 3:**
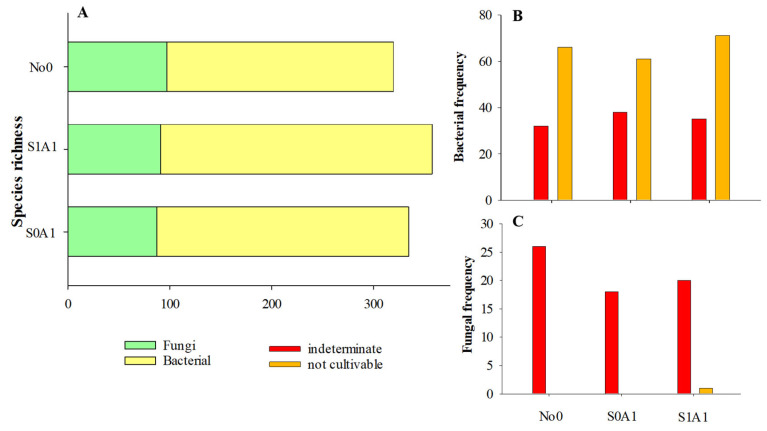
Frequency of cultivable, non-cultivable, and undetermined microbial species (richness) associated with the rhizosphere system of *Avena sativa*. (**A**) Species richness; (**B**) Bacterial frequency; (**C**) Fungal frequency. S0A1—*Avena sativa* cultivation in an area with 0.0 g ha^−1^ of sulfentrazone; S1A1—*A. sativa* cultivation in an area with a dose of 600 g ha^−1^ of sulfentrazone; No0—Typical soil of the Cerrado biome. Undetermined: species not yet identified at the species level by science; uncultivated: microorganisms previously identified in scientific studies but not yet characterized in the laboratory.

The number of species in the No0, S0A1, and S1A1 environments was significant. Species richness followed the ascending order S1A1 < S0A1 < No0, in which the implementation of *A. sativa* as a phytoremediator (S0A1) provided a modification of the pioneer community with an increase in frequency in 15 species and, when managed with herbicide, 38 species (S1A1) (Table 2, Figure 3).

The ecological explosions increased with greater bacterial richness intensity, presenting 74.5, 73.9, and 69.5% of bacteria in areas S1A1, S0A1, and No0, respectively. Fungal richness was 30.4, 26.0, and 25.5% in areas No0, S0A1, and S1A1, respectively (Figure 3A).

The frequency of bacteria with potential for herbicide degradation showed 9.6% of species unknown to science (undetermined) and 19.8% of non-cultivable bacteria in No0. Similar percentages were identified in the other environments: in S0A1, 11.9% of the bacteria corresponded to undetermined species and 19.1% to non-cultivable species, while in S1A1, these values were 9.8% and 19.9%, respectively (Figure 3A,B).

The frequency of undetermined fungi was 7.8%, 5.7%, and 5.6% in areas No0, S1A1, and S0A1, respectively. In area S1A1, a fungus identified as *Rozellomycotina* was present. *Incertae sedis* was observed (Figure 3A,C).

The abundance distribution among species was altered by soil management (S0A1 and S1A1), compared to the typical Cerrado soil (No0) (Table 2, Figure 3). Positive and negative correlations were observed between microbial orders for each treatment analyzed, indicating self-regulation mechanisms or homeostatic reactions that restore the existing relationships in the community (Supplementary Material S2).

Each treated area presented different levels of global diversity: 0.8 in No0, 0.7 in S1A1, and 0.5 in S0A1 (Table 2). In the absence of sulfentrazone and *Avena sativa* cultivation, an equitable action of the native community (No0) was observed. In the absence of herbicides, the planting of A. sativa resulted in a community that specialized in interacting with the plant, presenting a moderate diversity (0.5—Gini–Simpson Index). In the presence of sulfentrazone (S1A1), there was an increase in diversity (0.7—Gini–Simpson Index), indicating that the herbicide promoted a more uniform, equitable, and potentially specialized microbial community in the degradation of chemical molecules of sulfentrazone or secondary metabolites produced by the phytoremediation plant.

Soil management with *A. sativa* in areas with residual persistence of 0.053 ± 0.007 mg·kg^−1^ of sulfentrazone promoted significant changes in the structure of the microbial community, favoring specialized groups, in which there was a 68% increase in the activities of microorganisms related to the promotion of plant growth and productivity (Table 3).

*Avena sativa* presented a more functional community for nitrogen-fixing bacteria in area S0A1. In regions with sulfentrazone residues (S1A1), the occurrence of 79.2% of known species with atmospheric nitrogen fixation activity in the soil associated with *A. sativa* plants was observed, a value 39.14% higher compared to area No0 and 29.8% higher than area S0A1, respectively (Table 3). The most abundant species are *Bradyrhizobium* sp. (3.37%), *Methylobacterium* sp. (1.16%), and *Burkholderiaceae* (2.16%), which stood out. The abundance of these species can be up to 1.75 times higher in areas S1A1 compared to areas No0 or S0A1 (Table 4).

Bacterial and fungal species with nutrient solubilization activities were found in the soil (Table 3, Table 4 and Table 5). The typical Cerrado areas presented 56.9% of the microbial population with the capacity to solubilize at least one type of mineral, a value 7.2% higher than in the S0A1 treatment and 2.1% higher than in the S1A1 treatment (Table 3). Among the most abundant bacterial species, *Bradyrhizobium* sp., *Burkholderiaceae* indeterminate, *Rhodococcus* sp., and *Streptomyces* sp. (S1A1) stood out (Table 4). The abundance of *Rhodococcus* sp. was 16.6 times greater in the S1A1 areas compared to the S0A1 areas and was not detected in the No0 soil.

Among the fungi, *Articulospora* sp., *Aspergillus* sp., *Penicillium jensenii*, *Penicillium* sp., *Pleurophragmium* sp., *Talaromyces* sp., *Trichoderma longipilis*, and *Trichoderma strigosellum* were identified. The relative abundance of *Aspergillus* and *Trichoderma* spp. was 101 and 30 times higher in No0 than S0A1, which were more recurrent. *Penicillium jensenii* is closely linked to *A. sativa*, occurring 18.2 times more in association with or without herbicide. In contrast, the fungus *Articulospora* sp. was not detected in No0 soils and was 3.8 times more recurrent in S1A1 (Table 5).

The siderophore-producing species are ranked in Table 3, Table 4 and Table 5. The presence of the herbicide increased by 12.9% and 17.2% when compared to No0 and S0A1, respectively. The bacteria *Arthrobacter cupressi*, *Mycobacterium* sp., *Nocardia* sp., *Rhodococcus* sp., and the fungi *Aspergillus* sp., *Talaromyces* sp., *Trichoderma longipilis*, and *Trichoderma strigosellum* were the most abundant organisms among the species identified in the S1A1 treatments (Table 4 and Table 5).

The biocontrol activity developed by bacteria and fungi was more abundant in the S1A1 (91.8%) and S0A1 (48.1%) areas, differing from each other and the No0 treatment (43.7%). A total of 72.6%, 66.1%, and 54.8% of the microorganisms in S1A1, No0, and S0A1, respectively, can produce molecules with fungicidal action; 70.9%, 53.2%, and 64.55% with bactericidal action; 33.8%, 35.5%, and 33.6% with nematicidal action; and 41.9%, 37.1%, and 45.2% with insecticidal action (Table 3, Table 4 and Table 5). Among the most abundant organisms were *Methylobacterium* sp., *Nocardia* sp., *Streptomyces* sp., *Burkholderiaceae* indeterminate, *Aspergillus* sp., *Chaetomium* sp., *Chrysanthotrichum lentum*, *Epicoccum* sp., *Hannaella oryzae*, *Humicola repens*, *Papiliotrema laurentii*, *Penicillium jensenii*, *Talaromyces* sp., stand out. *Purpureocillium* sp., *Trichoderma longipilis,* and *Trichoderma strigosellum* (Table 4 and Table 5).

The capacity to produce plant hormones was observed in the species Arthrobacter cupressi, *Aspergillus* sp., *Bradyrhizobium* sp., *Burkholderiaceae* indeterminate, *Didymellaceae* (family indeterminate), *Methylobacterium* sp., *Mycobacterium* sp., *Nocardia* sp., *Penicillium jensenii*, *Penicillium* spp., *Sphingomicrobium lutea. Streptomyces* sp. *Trichoderma longipilis,* and *Trichoderma strigosellum,* which are in greater abundance in at least one of the treatment areas. Area S1A1 showed the most incredible abundance (62.5%), followed by the treatment without sulfentrazone (54.2%) and the typical Cerrado soil (58.3%).

The combined use of sulfentrazone and *A. sativa* may be an efficient strategy to increase microbial functionality, with a population growth of some bacterial species, such as *Lacisediminimonas* sp., *Pseudarthrobacter* sp., *Pedococcus* sp., and *Rhodococcus* sp., and fungal species, such as *Articulospora* sp., Filobasidium stepposum, *Hannaella oryzae*, *Plectosphaerella cucumerina*, and *Pleurophragmium* sp., which were not detected in the No0 samples, but had their growth enhanced when subjected to soil decontamination technology (Table 4 and Table 5).

The typical condition of the Cerrado (No0) presents good levels of functionality, although generally little explored by researchers, highlighting the positive impact of interventions in stimulating specific microbial processes.

## 4. Discussion

The results of this study highlight the potential of *Avena sativa* as an effective phytoremediation strategy for soils contaminated with sulfentrazone. Its ability to tolerate, accumulate, and promote the dissipation of the herbicide reveals a synergistic interaction with the soil microbiota, mediated by plant metabolization mechanisms and the activation of specialized microbial communities [31]. The reduction in contaminant concentration is associated with rhizospheric activity and the induction of degradative enzymes present in both the plant and the microorganisms, thereby decreasing the bioavailability of sulfentrazone to subsequent organisms [1].

Although the growth of *A. sativa* was affected by toxic stress, a high abundance of plant growth-promoting microorganisms was observed in treatment S1A1. Despite reports of a limited antioxidant system response to the presence of the herbicide, there was a reduction of approximately one-third in the activity of enzymes such as ascorbate peroxidase, catalase, and guaiacol peroxidase, as well as in lipid peroxidation [12]. These changes indicate an adaptive response to oxidative stress but impact the plant’s agronomic performance, particularly in biomass and grain yield. However, the species’ energy potential offsets agronomic losses, adding value to integrated remediation and bioenergy systems.

The persistence of sulfentrazone depends on soil properties [4] and edaphoclimatic variables, being more prolonged in winter—the preferred season for *A. sativa* cultivation [32]. The composition of the grass straw varies depending on genotype and planting season, allowing year-round cultivation [13]. Compounds such as p-coumarates and tricin, which are especially attractive from a biorefinery perspective, maintain acceptable levels even under abiotic stress, provided suitable varieties are adopted [13]. These findings reinforce the applicability of the species in breeding programs focused on bioenergy production and functional products.

Combining *A. sativa* with plant growth-promoting microorganisms stimulates soil enzymatic activity and improves remediation efficiency [33]. This biotechnological approach is more sustainable than conventional methods such as adsorption, reverse osmosis, and bioventing. Even with reduced plant biomass, *A. sativa* produced a significant amount of biogas, reinforcing its feasibility in integrated remediation and bioenergy systems. Modifying the microbial composition through consortia or adjusting herbicide dosage may optimize results, balancing decontamination capacity with commercial viability. Herbicide dose adjustment can be achieved through investments in application technology and integrated weed management, advocating for economically viable doses.

Management with *A. sativa* impacted the structure and functionality of the soil microbial community. Sulfentrazone increased microbial diversity and favored the emergence of degrading groups associated with biological nitrogen fixation, nutrient solubilization, and pathogen biocontrol. This ecological dynamic reflects microbial adaptation to a disturbed environment, with the succession of beneficial microorganisms absent from the pioneer community. Complementary research with *Crotalaria juncea* also indicates increased microbial activity and reduced residual fractions of sulfentrazone [1]. Strains such as *Aspergillus niger*, *Penicillium pinophilum*, *Trichoderma* sp., and *Bacillus subtilis* have also demonstrated tolerance to various herbicides, including sulfentrazone, and may complement phytoremediation systems [17].

In summary, management with *A. sativa* represents a viable, sustainable, and multifunctional alternative for the remediation of soils contaminated with sulfentrazone, integrating environmental, economic, and agronomic benefits. Future investigations should explore the role of uncultivable microorganisms and identify genes of biotechnological interest to maximize remediation efficiency. Additionally, further studies are needed to optimize management practices, explore specific microbial consortia, and assess the applicability of the technology across different edaphoclimatic contexts. Assessing the impacts on microbial diversity and ecosystem services is essential to consolidate this strategy as an integrated and sustainable solution.

## 5. Conclusions

This study demonstrates that *Avena sativa* can tolerate and promote the dissipation of sulfentrazone in the soil, reducing its residual concentration by 19.7%. This performance highlights the viability of the species as an efficient and sustainable tool for the remediation of soils contaminated by persistent herbicides.

The synergistic interaction between *A. sativa* and the soil microbiota promoted the activation of specialized microbial communities, enriching microbiological functionality and favoring the biodegradation of sulfentrazone. The relative abundance of growth-promoting microorganisms (68%) in the S1A1 treatment confirms the key role of this interaction in the success of phytoremediation.

Although sulfentrazone caused a significant reduction in growth (68.43%) and grain yield (60.0%), the energy potential of *A. sativa* biomass (340.6 m^3^ ha^−1^ of biogas) suggests its integration into phytoremediation and bioenergy systems, compensating for agronomic losses.

Management with *A. sativa* induced significant changes in the structure and functionality of the microbial community, favoring specialized groups such as *Bradyrhizobium* sp., *Rhodococcus* sp., and *Trichoderma* spp. These microorganisms perform critical functions such as nitrogen fixation and contaminant biodegradation, reinforcing the role of *A. sativa* as a modulator of ecological processes in the soil.

Integrating *A. sativa* into phytoremediation systems offers an environmentally sustainable approach, closing the remediation cycle with economic and ecological benefits. The technique combines decontamination efficiency with a lower environmental impact than conventional remediation methods.

However, it is important to recognize that both phytoremediation and bioremediation techniques have limitations. These processes generally require extended periods to reach desirable decontamination levels. They may be ineffective in soils with high contaminant concentrations, and their success heavily depends on edaphoclimatic factors such as soil type, temperature, and moisture. Furthermore, the large-scale use of remediating plants may demand extensive areas and specialized management, while introducing exogenous microorganisms can face adaptation challenges and competition with native microbiota. Therefore, improving these techniques requires an integrated approach and the development of biotechnological solutions to overcome these limitations and maximize their efficiency in different environmental contexts.

## Figures and Tables

**Table 1:** Growth of *Avena sativa* plants after 100 days of planting in areas decontaminated with sulfentrazone molecules.

Treatments	Height (cm)	Dry Mass (g)	PMG (g)	Productivity (Kg ha^−1^)	Ethanol Production (L ha^−1^)	Biogas Production (m^3^ ha^−1^)
S0A1	85.33 ^a^	134.88 ^a^	40.62 ^a^	2368.36 ^a^	710.5 ^a^	1079.0 ^a^
S1A1	51.83 ^b^	42.57 ^b^	24.79 ^b^	947.86 ^b^	284.4 ^b^	340.6 ^b^

S0A1—*Avena sativa* cultivation in an area with 0.0 g ha^−1^ of sulfentrazone; S1A1—*A. sativa* cultivation in an area with 600 g ha^−1^ of sulfentrazone. Means followed by the same lowercase letter in the columns did not differ statistically, according to Tukey’s test at 5% significance.

**Table 2:** Global ecological index of microbial diversity in an area of *Avena sativa* in soil with residues of the herbicide sulfentrazone.

Avena sativa				
	Unif ^1^	Riqueza	Dist. ^2^	Diver ^3^
S0A1	0.7 ^ns^	334 *	2.6 *	0.5 *
S1A1	0.7 ^ns^	357 *	2.7 *	0.7 *
No0	0.8 ^ns^	319 *	2.9 *	0.8 *

S0A1—*Avena sativa* cultivation in an area with 0.0 g ha^−1^ of sulfentrazone; S1A1—*A. sativa* cultivation in an area with 600 g ha^−1^ of sulfentrazone; No0—Typical soil of the Cerrado biome. ^1^ Uniformity: Pielou Index; Richness: Floor 1 Index; ^2^ Species distribution: Shannon Index. ^3^ Diversity: Gini–Simpson Index. Consecutive means “ns” did not differ statistically from each other, according to the Z test at 5% significance. The consecutive means “*” differed statistically from each other, according to the Z test at 5% significance.

**Table 3:** Relative abundance (%) of the microbiome with activity promoting plant growth and productivity in a phytoremediation area with *Avena sativa* in soil with residues of the herbicide sulfentrazone.

Table	Biological Nitrogen Fixation	Nutrient Solubility	Siderophores	Biocontrol	Hormones
S0A1	55.6 *	52.8 *	47.1 *	48.1 *	54.2 *
S1A1	79.2 *	55.7 *	56.9 *	91.8 *	62.5 *
No0	48.6 *	56.9 *	49.8 *	43.7 *	58.3 *

S0A1—*Avena sativa* cultivation in an area with 0.0 g ha^−1^ of sulfentrazone; S1A1—*A. sativa* cultivation in an area with 600 g ha^−1^ of sulfentrazone; No0—Typical soil of the Cerrado biome. Consecutive means * differed statistically from each other by the Z test at 5% significance.

**Table 4:** Relative abundance (%) of the bacterial microbiome has the highest percentage by area type and growth-promoting activities in *Avena sativa* plants used as a phytoremediator of sulfentrazone herbicide molecules.

Bacterial Species	Abundance (%)			Fitness				
	S0A1	S1A1	No0	FBN	Side	Bioc	Sol	Horm
*Arthrobacter cupressi*	0.71	0.1	1.77	-	+	-	-	+
*Baekduia* sp.	0.89	0.71	1.90	-	-	-	-	-
*Bradyrhizobium* sp.	2.48	3.37	2.75	+	-	-	+	+
*Ktedonobacter* sp.	0.16	0.13	1.17	-	-	-	-	-
*Lacisediminimonas* sp.	0.08	1.85	0.0	-	-	-	-	-
*Massilia* sp.	1.30	0.57	0.27	-	-	-	-	-
*Methylobacterium* sp.	0.66	1.16	0.35	+	-	+	-	+
*Mycobacterium* sp.	1.46	2.18	2.45	-	+	-	-	+
*Nocardia* sp.	0.57	1.15	0.15	-	+	+	-	+
*Paenarthrobacter* sp.	1.24	0.90	0.05	-	-	-	-	-
*Pedococcus* sp.	1.10	0.85	0.03	-	-	-	-	-
*Pseudarthrobacter* sp.	3.47	0.53	0.06	-	-	-	-	-
*Rhodococcus* sp.	0.20	3.31	0.00	-	+	-	+	-
*Sphingomicrobium lutea*	1.33	2.16	0.61	-	-	-	-	+
*Streptomyces* sp.	2.33	0.89	1.28	-	-	+	+	+
(Classe) *Alphaproteobacteria* indeterminate	0.82	1.07	1.41	-	-	-	-	-
(Classe) *Dormibacteria* uncultivated	0.10	0.10	2.41	-	-	-	-	-
(Familia) *Bryobacteraceae* indeterminate	0.50	0.89	2.09	-	-	-	-	-
(Familia) *Burkholderiaceae* indeterminate	0.50	2.13	0.3	+	-	+	+	+
(Familia)*Ktedonobacteraceae* indeterminate	0.12	0.22	1.11	-	-	-	-	-
(Familia) *Ktedonobacteraceae* uncultivated	0.10	0.10	1.86	-	-	-	-	-
(Familia) *Micrococcaceae* indeterminate	30.99	3.40	0.61	-	-	-	-	-
(Familia)*Solirubrobacteraceae* indeterminate	2.11	2.64	8.50	-	-	-	-	-
(Familia) *Steroidobacteraceae* uncultivated	0.48	0.62	1.94	-	-	-	-	-
(Familia)*Streptosporangiaceae* uncultivated	0.20	0.30	1.06	-	-	-	-	-
(Familia) *Xanthobacteraceae* indeterminate	3.51	3.39	4.88	-	-	-	-	-
(Familia) *Xanthobacteraceae* uncultivated	0.92	2.1	5.17	-	-	-	-	-
(Ordem) *Acidimicrobiales* indeterminate	0.50	0.6	1.43	-	-	-	-	-
(Ordem) *Acidobacteriales* uncultivated	0.1	0.5	3.05	-	-	-	-	-
(Ordem) *Chthoniobacterales* uncultivated	1.25	1.22	4.31	-	-	-	-	-

S0A1—*Avena sativa* cultivation in an area with 0.0 g ha^−1^ of sulfentrazone; S1A1—*A. sativa* cultivation in an area with 600 g ha^−1^ of sulfentrazone; No0—Typical soil of the Cerrado biome. (+) presence; (-) absence of activity.

**Table 5:** Relative abundance (%) of the fungal microbiome has the highest percentage by area type and growth-promoting activities in *Avena sativa* plants used as a phytoremediator of sulfentrazone herbicide molecules.

Fungi Species	Abundance (%)			Fitness			
	S0A1	S1A1	No0	Bioc	Side	Horm	Sol
*Absidia* sp.	0.9	0.0	3.5	-	-	-	-
*Arachnion album*	0.0	0.0	1.7	-	-	-	-
*Articulospora* sp.	0.5	1.9	0.0	-	-	-	+
*Aspergillus* sp.	0.1	0.1	10.1	+	+	+	+
*Chaetomium* sp.	2.8	13.3	0.1	+	-	-	-
*Chrysanthotrichum lentum*	0.1	0.2	4.4	+	-	-	-
*Cladosporium* sp.	13.1	1.9	3.7	-	-	-	-
*Didymellaceae* (family indet).	10.0	13	1.5	-	-	+	-
*Epicoccum* sp.	3.0	17.1	1.3	+	-	-	-
*Filobasidium stepposum*	2.2	0.5	0.0	-	-	-	-
*Fusarium chlamydosporum*	0.8	0.8	0.2	-	-	-	-
*Fusarium oxysporum*	27.7	18.8	17.4	-	-	-	-
*Hannaella oryzae*	1.9	0.1	0.0	+	-	-	-
*Humicola repens*	5.2	1.0	1.4	+	-	-	-
*Papiliotrema laurentii*	1.3	0.2	0.2	+	-	-	-
*Penicillium jensenii*	3.7	2.5	0.2	+	-	+	+
*Penicillium* spp.	5.6	4.4	9.8	+	-	+	+
*Plectosphaerella cucumerina*	1.0	0.1	0.0	-	-	-	-
*Pleurophragmium* sp.	1.3	0.55	0.0	-	-	-	+
*Purpureocillium* sp.	0.3	0.3	1.1	+	-	-	-
*Pyrenochaetopsis leptospora*	2.9	0.9	0.2	-	-	-	-
*Talaromyces* sp.	0.3	1.1	0.9	+	-	-	+
*Trichoderma longipilis*	0.2	0.3	6.0	+	+	+	+
*Trichoderma strigosellum*	0.3	0.4	2.3	+	+	+	+
*Fungi indeterminate*	0.3	1.4	1.2	-	-	-	-

S0A1—*Avena sativa* cultivation in an area with 0.0 g ha^−1^ of sulfentrazone; S1A1—*A. sativa* cultivation in an area with 600 g ha^−1^ of sulfentrazone; No0—Typical soil of the Cerrado biome. (+) presence; (-) absence of activity.

## Data Availability

The original contributions presented in this study are included in the article/Supplementary Material. Further inquiries can be directed to the corresponding author.

## Supplementary Materials

The following supporting information can be downloaded at: https://www.mdpi.com/article/10.3390/jox15030087/s1, Supplementary Material S1: Residual persistence of sulfentrazone in soils in the absence (S1A0) and presence (S1A1) of *Avena sativa* after 80 days of planting in areas contaminated with 600 g ha^−1^ of sulfentrazone. The values presented in the figures are given as means ± standard errors. Supplementary Material S2: Neural correlation network of microbial orders associated with *Avena sativa* in residual sulfentrazone areas. S0A1—*Avena sativa* cultivation in an area with 0.0 g ha^−1^ of sulfentrazone; S1A1—*A. sativa* cultivation in an area with 600 g ha^−1^ of sulfentrazone; No0—Typical soil of the Cerrado biome. The neural correlation networks were clustered using the Pearson procedure with a significance cutoff (>−0.6), orange lines with negative correlation, and blue lines with positive correlation (>0.6). Microbial orders: 1—*Acetobacterales*; 2—*Agaricales*; 3—Indeterminate_A; 4—*Azospirillales*; 5—*Baltobacterales*; 6—*Bryobacterales*; 7—*Burkholderiales*; 8—*Capnodiales*; 9—*Caulobacterales*; 10—Indetermiando_B; 11—*Chaetosphaeriales*; 12—*Chaetothyriales*; 13—*Chthoniobacterales*; 14—*Coniochaetales*; 15—*Deinococcales*; 16—*Dormibacterales*; 17—*Enterobacterales*; 18—*Eurotiales*; 19—*Philobasidiales*; 20—*Gaiellales*; 21—*Gemmatales*; 22—*Gemmatimonadales*; 23—*Glomerellales*; 24—*Helotiales*; 25—*Hypocreales*; 26—*Isosphaerales*; 27—*Ktedonobacterales*; 28—*Limnocylindrales*; 29—*Magnaporthales*; 30—*Mortierellales*; 31—*Mucorales*; 32—*Mycobacteriales*; 33—*Pleosporales*; 34—*Propionibacteriales*; 35—*Rhizobiales*; 36—*Solirubrobacterales*; 37—*Sordariales*; 38—*Sphingomonadales*; 39—*Spizellomycetales*; 40—*Sporidiobolales*; 41—*Steroidobacterales*; 42—*Streptomycetales*; 43—*Streptosporangiales*; 44—*Tepidisphaerales*; 45—*Tremellales*; 46—*Tumebacillales*; 47—*Venturiales*; 48—*Vicinamibacterales*.
