# Supplementary material for: Avena sativa as a Multifunctional Tool for Phytoremediation and Bioenergy Production in Sulfentrazone Contaminated Soils
Source: J Xenobiot. 2025 Jun 4;15(3):87. doi: 10.3390/jox15030087 (PMC12193893; doi:10.3390/jox15030087)
Supplement: Supplementary file 1 [file jox-15-00087-s001.zip › jox-3594354-supplementary.pdf]

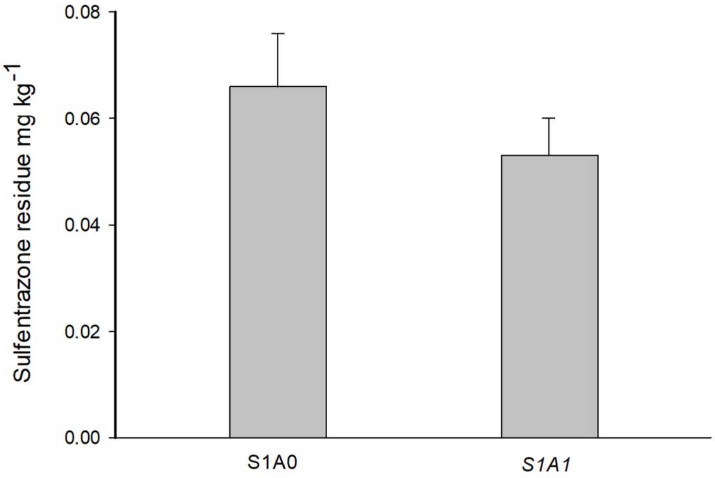

Supplementary Material S1: Residual persistence of sulfentrazone in soils in the absence (S1A0) and presence (S1A1) of *Avena sativa* after 80 days of planting in areas contaminated with 600 g ha^{-1} of sulfentrazone. The values presented in the figures are given as means ± standard errors.

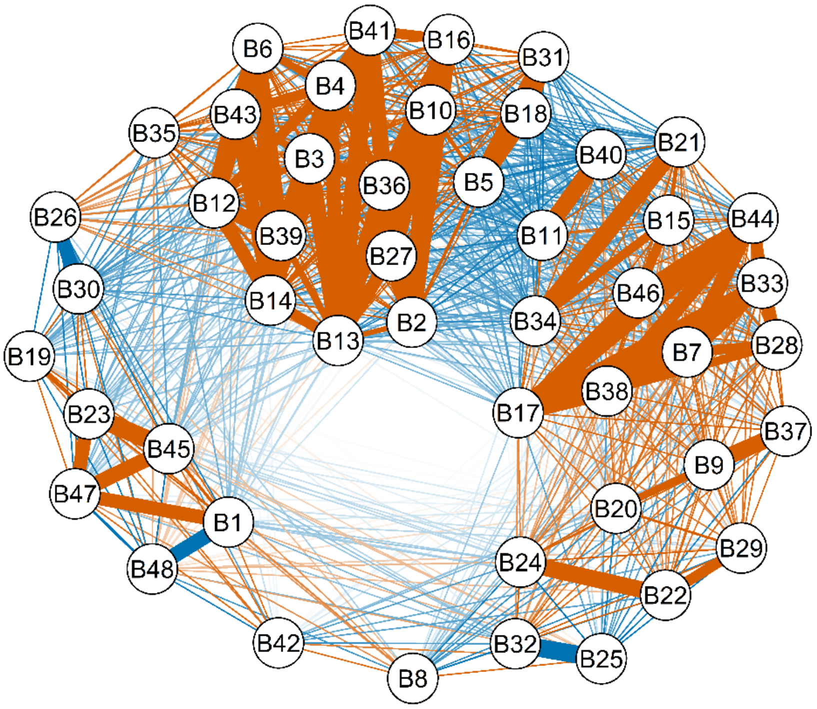

Supplementary Material S2: Neural correlation network of microbial orders associated with *Avena sativa* in residual sulfentrazone areas. S0A1—*Avena sativa* cultivation in an area with 0.0 g ha^{-1} of sulfentrazone; S1A1—*A. sativa* cultivation in an area with 600 g ha^{-1} of sulfentrazone; No0—Typical soil of the Cerrado biome. The neural correlation networks were clustered using the Pearson procedure with a significance cutoff (> -0.6), orange lines with negative correlation, and blue lines with positive correlation (>0.6). Microbial orders: 1—*Acetobacterales*; 2—*Agaricales*; 3—Indeterminate_A; 4—*Azospirillales*; 5—*Baltobacterales*; 6—*Bryobacterales*; 7—*Burkholderiales*; 8—*Capnodiales*; 9—*Caulobacterales*; 10—Indetermiando_B; 11—*Chaetosphaeriales*; 12—*Chaetothyriales*; 13—*Chthoniobacterales*; 14—*Coniochaetales*; 15—*Deinococcales*; 16—*Dormibacterales*; 17—*Enterobacterales*; 18—*Eurotiales*; 19—*Philobasidiales*; 20—*Gaiellales*; 21—*Gemmatales*; 22—*Gemmatimonadales*; 23—*Glomerellales*; 24—*Helotiales*; 25—*Hypocreales*; 26—*Isosphaerales*; 27—*Ktedonobacterales*; 28—*Limnocylindrales*; 29—*Magnaporthales*; 30—*Mortierellales*; 31—*Mucorales*; 32—*Mycobacteriales*; 33—*Pleosporales*; 34—*Propionibacteriales*; 35—*Rhizobiales*; 36—*Solirubrobacterales*; 37—

Sordariales; 38—*Sphingomonadales*; 39—*Spizellomycetales*; 40—*Sporidiobolales*; 41—*Steroidobacterales*; 42—*Streptomycetales*; 43—*Streptosporangiales*; 44—*Tepidisphaerales*; 45—*Tremellales*; 46—*Tumebacillales*; 47—*Venturiales*; 48—*Vicinamibacterales*.
